# Epigenetic modifications of caveolae associated proteins in health and disease

**DOI:** 10.1186/s12863-015-0231-y

**Published:** 2015-06-26

**Authors:** Jin-Yih Low, Helen D. Nicholson

**Affiliations:** Department of Anatomy, Otago School of Medical Sciences, University of Otago, P.O. Box 913, Dunedin, 9054 New Zealand

**Keywords:** Caveolae, Epigenetic, micro-RNA, Promoter methylation, Histone acetylation, PTRF, Caveolin-1, Caveolin-2, 5-AZA, Trichostatin-A

## Abstract

Caveolae are small, “omega-shaped” invaginations at the plasma membrane of the cell which are involved in a variety of processes including cholesterol transport, potocytosis and cell signalling. Within caveolae there are caveolae-associated proteins, and changes in expression of these molecules have been described to play a role in the pathophysiology of various diseases including cancer and cardiovascular disease. Evidence is beginning to accumulate that epigenetic processes may regulate the expression of these caveolae related genes, and hence contribute to disease progression. Here, we summarize the current knowledge of the role of epigenetic modification in regulating the expression of these caveolae related genes and how this relates to changes in cellular physiology and in health and disease.

## Introduction

Caveolae are small specialized “cave-like” microdomains at the plasma membrane that function as trafficking vesicles and are involved in organization of signal transduction. Caveolae are present in most tissues and are particularly abundant in cardiac, continuous endothelial and epithelial cells, as well as fat cells [1–3]. Within caveolae are caveolae associated proteins; caveolin-1 (CAV1) [4], caveolin-2 (CAV2) [5], caveolin-3 (CAV3) [6], Cavin-1 (also know as polymerase-1 and transcript release factor) (PTRF) [7], Cavin-2 [8], Cavin-3 [9] and Cavin-4 [10], which are important for the formation and maintenance of the caveolar structure.

CAV1 is a 22 kDa protein which is the principal substrate of src kinase [11] and appears as a filament-like structure at the plasma membrane [12]. CAV1 is expressed in a wide range of tissues with the highest expression in smooth muscle cells, adipocytes, fibroblasts and endothelial cells [13]. CAV1 plays an important role in the formation of caveolae; if cells lack CAV1, no caveolae are observed [14] while, restoration of CAV1 expression results in the *de novo* formation of caveolae [15, 14]. *CAV1* knock out mice demonstrate a variety of physiological defects including reduced renal calcium reabsorption and vascular and metabolic abnormalities [16–18]. CAV1 is also reported to be involved in diseases such as cancer, cardiovascular disease and diabetes (for review see [19]).

CAV2 is a 20 kDA protein found abundantly in white adipose tissue [5]. Expression of CAV2 is independent of caveolae formation, however, co-expression of CAV2 with CAV1 results in more abundant invaginations and more uniform caveolae formation [20, 21]. Thus while CAV2 may not be essential, it plays a supporting role in modulating the biogenesis of caveolae. CAV2 is expressed concurrently with CAV1 and can undergo hetro-oligomerization with CAV1 [22]. In addition, CAV2 has been shown to interact with CAV3 in cardiac muscle cells [23]. *CAV2* knock out mice have normal distribution of caveolae but display a variety of lung disorders [21].

CAV3 has a molecular weight of 18–20 kDA and is 85 % similar to CAV1 [6]. It is predominantly expressed in muscle cells [6]. CAV3 co-immunoprecipitates with dystrophin, suggesting that dystrophin and CAV3 can exist as a discrete complex [24]. In embryonic fibroblasts derived from caveolae-null mice, restoration of *CAV3* successfully restores the formation of caveolae [25]. *CAV3* knock out mice show a loss of caveolae at the sarcolemma (but not endothelial cells), exclusion of dystrophin-glycoprotein complexes from the lipid rafts, abnormalities of the T-tubule system, insulin resistance and instability of the insulin receptor in skeletal muscle [26, 27]. Similarly, analysis of cardiac muscle from *CAV1* knock out mice demonstrates a loss of caveolae in the cardiac endothelial cells but not cardiac myocytes, however the opposite observation was seen in *CAV3* knock out mice [28]. Only in *CAV1 CAV3* double knock out mice were caveolae completely abolished in both cell types [28]. This suggests that *CAV3* can compensate for *CAV1* allowing caveolae formation in cardiac myocytes, providing some functional redundancy [28].

*PTRF* was cloned in 1998 and was first described to be involved in RNA transcription machinery [29, 30]. PTRF is a resident protein in caveolae [31] and is widely expressed in a range of tissues, with highest expression in adipocytes, cardiac and skeletal muscles and osteoblasts [32]. The functional role of PTRF in caveolae formation has only recently been described. Loss of PTRF is accompanied by reduced numbers of caveolae [33, 34]. Re-expression of *PTRF* in cell lines that have reduced or lack PTRF results in caveolae formation [35, 7]. *PTRF* knock out mice lack caveolae and demonstrate glucose intolerance and disorders of the lungs and cardiovascular system [34, 36–39].

Structurally, Cavin-2 is ~ 20 % similar to PTRF [40]. Although down-regulation of Cavin-2 in turn causes reduced PTRF and CAV1 expression (hence reduced caveolae number), suggesting the interdependency between these 3 molecules [8], the expression of Cavin-2 alone does not alter the number of caveolae [40, 41]. However, the expression of Cavin-2 induces tube-like morphological changes to caveolae [40]. Cavin-3 is reported to be associated with CAV1 during caveolae budding [9]. The process of caveolae budding and trafficking of caveolae-associated vesicles along the microtubules is greatly impaired in the absence of Cavin-3, suggesting a role of Cavin-3 in intracellular transport mechanisms [9]. Cavin-4 is only present in muscle cells and is a cytosolic protein that is able to interact with Cavin-2. Cavin-4 has been demonstrated to be important in cardiac dysfunction where Cavin-4 is able to modulate the Rho/ROCK pathway that is important for cardiac muscle biogenesis [42].

Changes in the expression of the caveolae related proteins are associated with disease. For example, expression of CAV1, CAV2 and PTRF is dysregulated in prostate and breast cancer [33, 43]. Furthermore, other health issues such as cardiovascular disease, inflammation and abnormal insulin signaling are associated with changes in these proteins [44, 39]. However, what causes the change in expression of these caveolae related molecules is unknown. Potentially, these changes may be related to epigenetic or micro-RNA (miRNA) mechanisms that act upstream of the genes. This review brings together the current evidence for epigenetic regulation of these genes and thus, presence of caveolae. As there are limited data, or no evidence, published on CAV3, Cavin-2, Cavin-3 and Cavin-4, this review will focus on CAV1, CAV2 and PTRF.

## Review

### Evidence for epigenetic changes related to *CAV1*, *CAV2* and *PTRF*

Epigenetics involves the study of the changes in gene expression that are independent of any changes in DNA sequences. There are two main mechanisms under the umbrella of epigenetics; DNA methylation, which involves the methylation of the promoter region of the gene and histone deacetylation which involves structural changes of the chromatin. Importantly epigenetic changes can be reversed with the use of chemical agents [45].

DNA promoter hypermethylation involves the modification of cytosine residues in the CpG dinucleotides to form 5-methylcytosine through covalent addition of a methyl group by the enzyme, DNA methyltransferase. In the mammalian genome, CpG dinucleotides are unevenly distributed to form short sequences that have high densities of CpG dinucleotides known as CpG islands (CpGi) [46], within the promoter region of the genome. Gene promoters which have their CpGi methylated are transcriptionally inactive as the methyl groups block the promoter region from being accessed by transcriptional elements [45]. Chemical agents such as 5-AZA-2′-deoxycytidine (5-AZA) have been reported to reverse DNA promoter hypermethylation [47–49].

More recent studies suggest that methylation can also occur in non-CpGi rich areas in the promoter region to silence gene expression. These regions have a lesser density of CpGi and are normally situated around 2 kb from the regular CpGi rich regions, and have been named CpGi shores [50, 51]. Hypermethylation at the CpGi shore appears to have a critical role in regulating gene expression [50]

Allfrey *et al.* [52] described that for gene expression to take place, the **ε**-amino group in the lysine residue of the histone cores must be acetylated by histone acetyltransferases (HATs). Histone deacetylases (HDACs) cancel the effect of HATs by removing acetyl groups from the lysine residue in histone cores. The removal of acetyl groups by HDACs restores the positive charges on lysine residues. This causes the histone tails to coil tightly to the DNA leading to transcriptional inactivation as the transcriptional machinery is unable to access the DNA [52, 53]. The use of Trichostatin-A (TSA), a microbial metabolite capable of inhibiting HDACs, was first described in 1995 and has been used to re-express genes which are inactivated by histone deacetylation [54]. Gene transcription is restored when lysine residues in the histone tails are acetylated through inactivation of HDACs by TSA [54].

To date, most of the reports of epigenetic effects on *CAV1* are related to DNA methylation and in the context of cancer (Table 1). It is suggested that the 5′ promoter of *CAV1* is methylated in human breast cancer cell lines, MDA-MB-231, MCF7 and T-47D but not in normal human mammary epithelial cells [55, 43]. Studies of clinical tissues have shown that breast cancer tissues have hypermethylation of the *CAV1* promoter accompanied by down-regulation of *CAV1* expression when compared to adjacent normal breast tissues [56, 57]. Furthermore, *CAV1* promoter hypermethylation is significantly associated with the histopathological grade of the tumor [56].

**Table 1:** Involvement of *CAV1* epigenetic modification in cancers

Cancer types	Promoter hypermethylation	Histone modification	*CAV1* expression	Changes in physiology and pathophysiology
Breast	[55]	[59]	Down-regulated	Decreased migration after 5-AZA and TSA treatment [59]
	[57]			
	[43]			
	[56]			
	[59]			
	[58]			
Prostate	[62]		Down-regulated	Not described
	[64]			
	[63]			
Bladder	[68]		Down-regulated	Not described
Ovarian	[71]	[71]	Down-regulated	Over-expression of *CAV1*, reduced colony formation and increased apoptosis in ovarian cancer cell line [71]
Lung	[70]		Down-regulated	Over-expression of *CAV1*, reduced colony formation in lung cancer cell line [70]
Colorectal	[72]		Down-regulated	Not described
	[73]			
	[74]			
Liver	[75]		Down-regulated	Not described
	[77]			

Nodal metastasis has been reported to be associated with *CAV1* hypermethylation [58]. It has been suggested that inactivation of *CAV1* through hypermethylation drives the spread of breast cancer to the lymph nodes [58]. Treatment of breast cancer cell lines with 5-AZA successfully increases both *CAV1* mRNA and protein [43, 59]. However, in a subtype of breast cancer, inflammatory breast cancer (IBC), *CAV1* is reported to be hypomethylated resulting in overexpression of *CAV1* [60]. Therefore it may suggest that different breast cancer subtypes may have different changes in epigenetic regulation of *CAV1*.

Recent evidence suggests that CpGi shores are involved in regulation of *CAV1* expression. Treatment with a DNA methyltransferase inhibitor induces expression of *CAV1* through demethylation of CpGi shores in breast cancer cell lines that have low *CAV1* expression (even though the CpGi rich promoters are hypermethylated) [61]. CpGi shores are reported to be hypermethylated in less aggressive breast cancer cells whereas hypomethylation of CpGi shores is observed in highly aggressive breast cancer cells [61].

In prostate cancer *CAV1* is down-regulated and this is accompanied by promoter hypermethylation of CpGi sites at the 5′ promoter region of *CAV1* [62, 63]. Bisulfite sequencing suggests promoter hypermethylation may be a mechanism for down-regulation of *CAV1* gene expression [64]. However, loss of CAV1 expression was observed in androgen dependent LNCaP cells while increased expression occurred in PC3 cells and tissues from advanced cancer [33]. Thus, expression of *CAV1* may be lost in the early stages of prostate cancer and promote cancer cell proliferation and survival, but up-regulated at late stages of prostate cancer to favor metastasis, inhibit apoptosis and promote multi-drug resistance [65]. A possible explanation for the conditional role of *CAV1* as both tumor promoter and suppressor is the interaction of CAV1 with other effector molecules that may directly or indirectly interact with or affect CAV1’s function [65]. Possible examples are Mgat5 and galectin-3. In early stages of prostate cancer, the expression of CAV1 is lost and the expression of Mgat5 and galectin-3 is at low levels. However, in later stages of prostate cancer, expression and formation of Mgat5/galectin-3 lattices may stimulate and elevate the expression of CAV1 through phosphorylation, resulting in up-regulated CAV1 expression in advanced prostate tumours [65–67].

Promoter methylation is also seen in a variety of other cancers and appears to be cell specific in a given organ (Table 1). For example, *CAV1* promoter methylation is seen in undifferentiated small cell and squamous cell carcinoma but not in transitional cell [68] or primary adenocarcinomas and signet ring cell carcinomas of the bladder [69]. Similar cell specificity is seen in lung cancers [70].

Treatment with 5-AZA has been shown to restore *CAV1* expression in some cancers confirming hypermethylation. *CAV1* expression is down-regulated in ovarian cancer cell lines but expression can be restored by treating the cells with 5-AZA [71]. *CAV1* promoter hypermethylation has also been reported in sporadic colorectal cancer [72] and re-expression of *CAV1* was observed in colon cancer cell lines after 5-AZA treatment [73, 74].

**Fig. 1:**
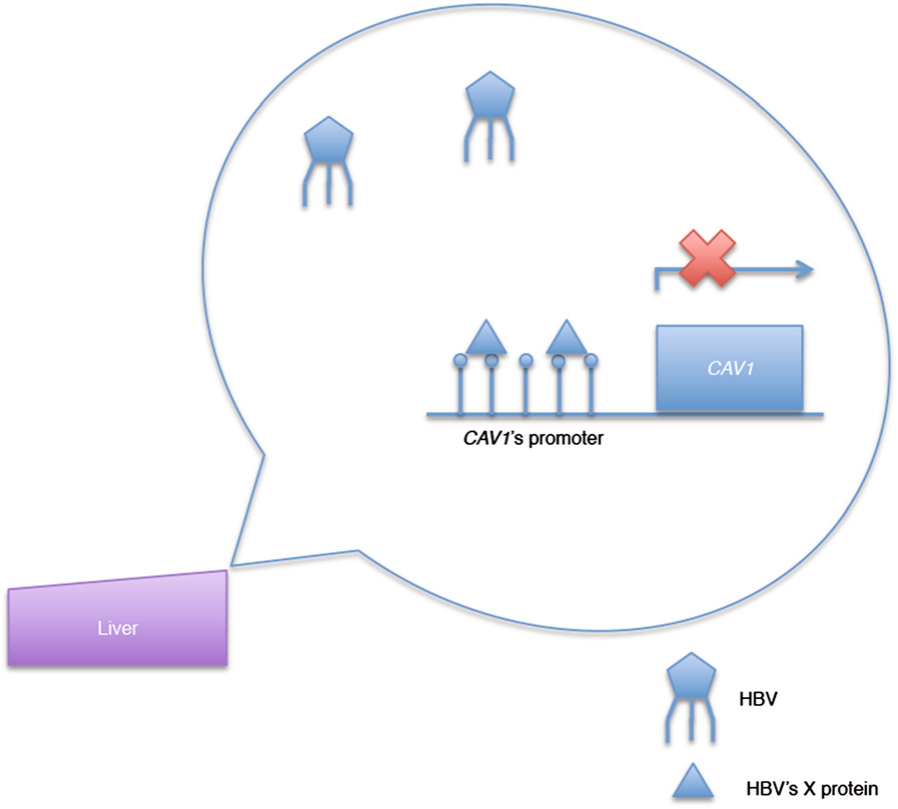
Effect of HBV’s X protein on *CAV1* expression. HCC samples infected with HBV demonstrate decreased *CAV1* expression. This effect is due to the promoter hypermethylation of *CAV1* by HBV’s X protein, causing transcriptional silencing of *CAV1*

Promoter hypermethylation of *CAV1* is also seen in hepatocellular carcinoma (HCC) cell lines [75] and HCC tissues and is accompanied by reduced expression of *CAV1* [75]. Further, 5-AZA treatment causes up-regulated *CAV1* expression in hepatoma cells [75]. One of the risk factors for hepatocellular carcinoma (HCC) is exposure to Hepatitis B Virus (HBV) and in particular to HBV’s X protein. This protein is able to promote tumorigenesis through activation of signaling pathways, growth factors and oncogenes. Furthermore, HBV’s X protein inactivates negative growth regulators such as p53 to favor metastasis [76, 77]. Interestingly, HCC samples that are infected with HBV show significant suppression of *CAV1* expression through hypermethylation of *CAV1*’s promoter [77], due to the hypermethylation effect of HBV’s X protein on *CAV1*’s promoter (Fig. 1) [77].

Other than promoter hypermethylation, histone modification has also been reported as a mechanism to silence *CAV1* expression. In ovarian cancer cell lines, treatment with TSA up-regulates *CAV1* [71] and in breast cancer cell lines, TSA treatment results in a 35 fold increase in *CAV1* expression [59].

**Fig. 2:**
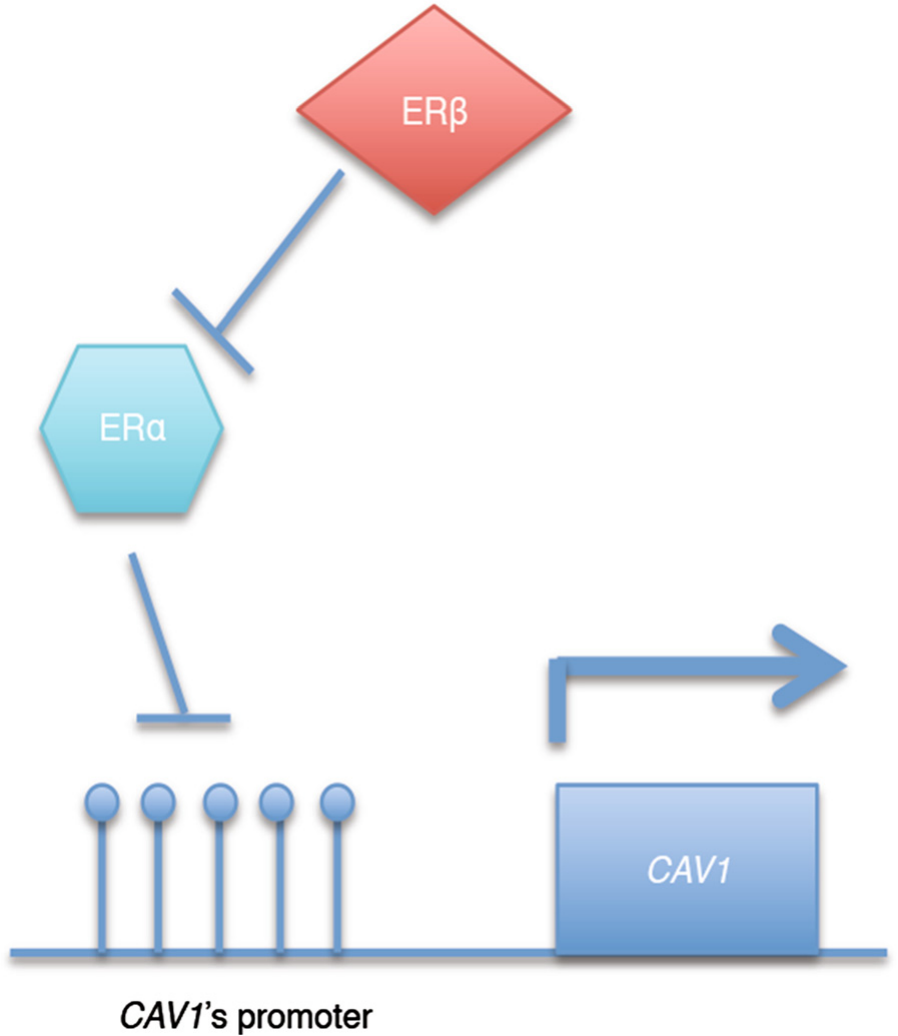
The mechanism of action of ER isoforms on *CAV1* expression. Over-expression of ERα leads to down-regulation of *CAV1* expression through epigenetic mechanisms. However, the co-expression of ERβ inhibits the effect of ERα, resulting in removal of the transcriptional suppression activity of ERα

Estrogen receptors alpha (ERα) and beta (ERβ) are expressed in neuronal cells [78, 79]. Ectopic expression of ERα in SK-N-MC neuronal cells leads to epigenetic silencing of *CAV1* (and down-regulation of CAV1) while treatment with 5-AZA and TSA results in partial restoration of *CAV1* expression. However, when ERβ is co-expressed with ERα in SK-N-MC cells, the effect on *CAV1* is abolished, suggesting ERβ counteracts the effect of ERα on *CAV1* down-regulation through an epigenetic pathway (Fig. 2) [80]. However, the exact molecular mechanism is not well understood and this observation may be due to a direct ERα targeting effect or indirect silencing of *CAV1* through ectopic expression of ERα. In neuronal cells that over-express ERα, *CAV2* expression is also down-regulated. 5-AZA treatment results in re-expression of *CAV2*, but TSA treatment has no effect [80]. This suggests that ERα is able to silence *CAV2* through DNA promoter methylation but not histone modification, suggesting another level of regulation.

To date, there are limited data describing the epigenetic regulation of *PTRF. PTRF* is down-regulated in breast cancer cell lines and tissues and this is related to promoter hypermethylation since *PTRF* was successfully restored through 5-AZA treatment [43].

### Summary of epigenetics and caveolae related genes

There is growing evidence of a role of epigenetic mechanisms in regulating *CAV1*, particularly in cancer (Table 1). These effects appear to be cell type specific and different epigenetic mechanisms may be involved in cells from different tissues. There is still limited knowledge on how epigenetics may regulate other caveolae related genes (*CAV2*, *CAV3* and *PTRF*).

### Evidence of microRNA regulation of *CAV1*, *CAV2* and *PTRF*

MicroRNAs (miRNAs) are able to regulate target transcription and hence protein expression through binding to the 3′-untranslated region of the matching target mRNA [81, 82]. These small nucleotides have been reported to be widely involved in physiological and pathophysiological processes such as apoptosis [83], cellular differentiation [84] and oncogenesis [85].

miRNAs have been shown to act as both tumor promoters and suppressors. Evidence suggests that miRNA-133a may act as an upstream regulator of *CAV1* expression in head and neck squamous cell carcinoma (HNCC) as the expression of miRNA-133a is down-regulated while *CAV1* is up-regulated in HNCC [86]. Luciferase reporter assays showed that miRNA-133a interacts directly with *CAV1* mRNA and transfection with a mirRNA-133a mimic results in down-regulated CAV1 expression [86].

**Fig. 3:**
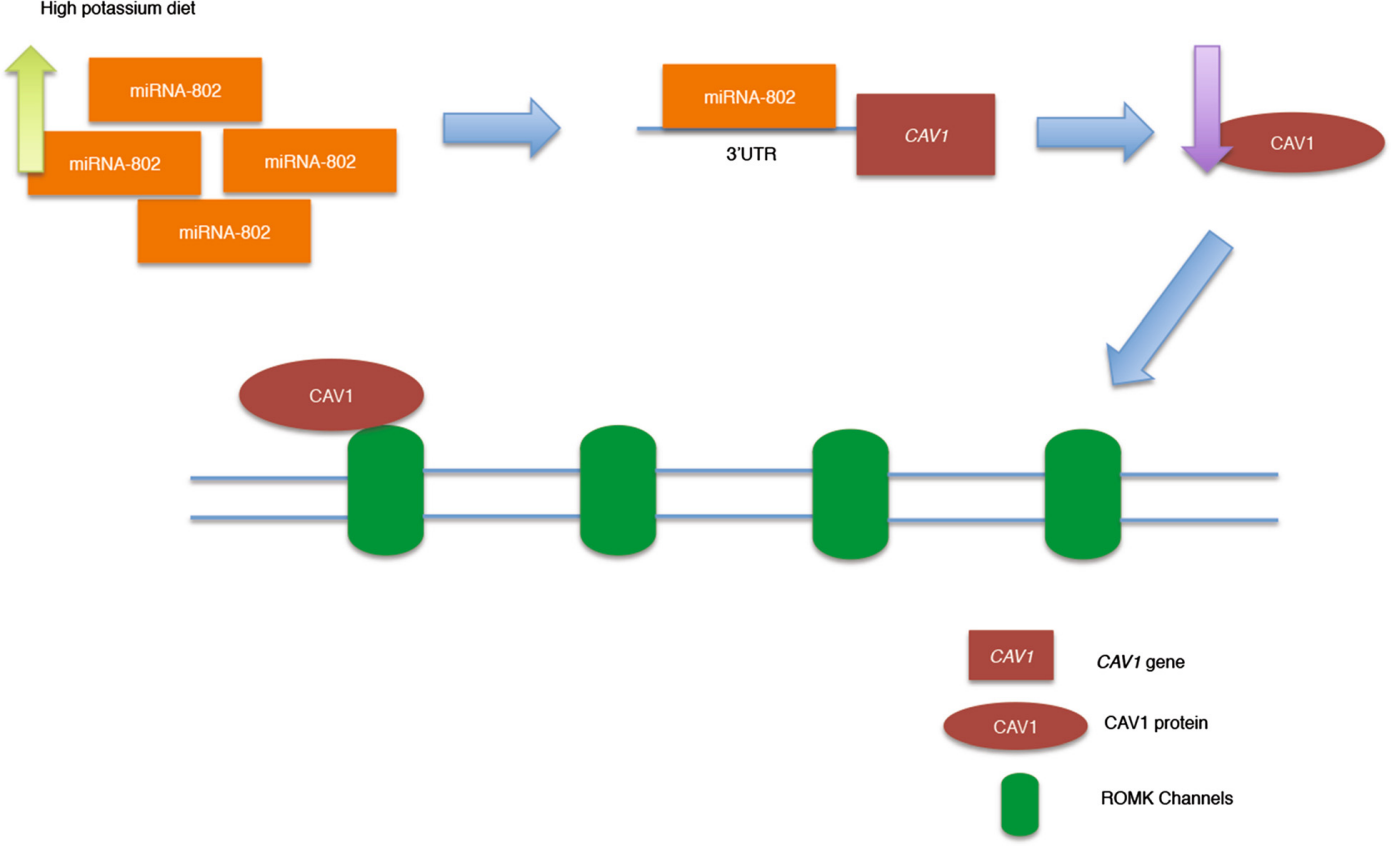
The role of dietary potassium in regulating miRNA-802 and CAV1 expression in the kidney. A diet high in potassium results in up-regulation of miRNA-802 which down-regulates *CAV1*’s expression by binding to 3′UTR of *CAV1*. Interaction of CAV1 with the N-terminus of the ROMK channels down-regulates these ion channels in the distal nephron, down-regulation of *CAV1* results in up-regulation of the ROMK channels

*In vivo*, a diet high in potassium results in increased expression of renal outer medullary potassium (ROMK) channels, an effect thought to be mediated by up-regulation of miRNA-802 [87]. The 3′-untranslated region (UTR) of *CAV1* contains sequences that allow direct interaction with miRNA-802. CAV1 inhibits ROMK channel activity by interacting with the N-terminus of ROMK channels [87]. When potassium increases, up-regulation of miRNA-802 occurs which down-regulates *CAV1*’s expression by binding to the 3′UTR of *CAV1*. As CAV1 is able to interact with the N-terminus of the ROMK channels (to down-regulate its expression) down-regulation of *CAV1* by miRNA-802 results in up-regulation of the ROMK channels (Fig. 3) [87].

In obese mice, miRNA-103 and 107 are up-regulated and may contribute to impaired glucose homeostasis [88]. Knocking down both miRNAs results in an improved response to insulin and glucose homeostasis [88]. CAV1 regulates insulin signaling [89] and *CAV1* is reported to be a target for both miRNA-103 and 107 [88]. Knocking down both miRNAs results in up-regulation of CAV1 [88] and a stabilization of insulin receptors and a responsive insulin signaling mechanism [88]. The findings suggest the potential use of miRNA-103 and 107 as therapeutic targets in treating diabetes and obesity.

*CAV1* has been shown to be a direct target for miRNA-199a-5p in the context of tissue fibrosis of several organs (liver, kidney, lungs) [90]. Up-regulation of miRNA-199a-5p in these tissues results in down-regulation of CAV1 [90]. Interestingly, TGF-β, a factor involved in fibrosis, induces the expression of miRNA-199a-5p, which in turn causes the down-regulation of CAV1 in these tissues [90]. In porcine adipocytes, there is a high expression of miRNA-199a-5p [91]. Over-expression of miRNA-199a-5p increases proliferation of pre-adipocytes and inhibits the deposition of lipid in adipocytes [91]. CAV1 has been shown to be involved in lipogenesis [92, 17] and potentially miRNA-199a-5p may play a role in controlling proliferation of adipocytes, partly through regulating the expression of *CAV1*.

In porcine kidney epithelial (PK15) cells, miRNA-124 has been shown to directly interact with *CAV1*. In these cells over-expression of miRNA-124 reduces *CAV1* expression at both mRNA and protein levels, thus reducing caveolae density and is associated with reduction in pathogen uptake [93]. Therefore, expression of miRNA-124 is proposed to be an important event that inhibits invasion of pathogens in the kidney through down-regulation of *CAV1*, and hence caveolae [93].

Docosahexaenoic acid (DHA) has been reported to modulate the transcriptome of miRNAs in lipid metabolism [94]. Exposure to DHA significantly increases the expression of miRNA-192 in enterocytes and *CAV1* is predicted to be a target for miRNA-192. Over-expression of miRNA-192, results in reduced *CAV1* expression [94]. However, the biological significance of this relationship is not yet known.

Expression of miRNA-199a-3p has been reported to be critical in promoting proliferation and survival of endothelial and breast cancer cells. *CAV2* has been shown to be a target of miRNA-199a-3p [95] with over-expression of *CAV2* inhibiting the effect exerted by miRNA-199a-3p in promoting proliferation, survival and sensitivity of cancer cells to anticancer drugs [95]. The interaction between miRNA-199a-3p and *CAV2* may provide an interesting target for intervention in cancer.

Loss of miRNA-218 and up-regulation of *CAV2* have been observed in renal cell carcinoma (RCC) [96]. Over-expression of miRNA-218 and knocking down *CAV2* significantly inhibits cellular proliferation, migration and invasion of RCC [96]. Gene expression studies reveal *CAV2* to be regulated by miRNA-218. It has been suggested that miRNA-218 acts as tumor suppressor by regulating *CAV2*, possibly through the focal adhesion pathway in RCC [96].

Interestingly, it has been shown that intestinal Salmonella infection is associated with miRNA-29a and CAV2. *CAV2* has been shown to be a direct target for miRNA-29a [97]. Infection with Salmonella causes up-regulation of miRNA-29a, which in turn results in down-regulation of *CAV2* and this is associated with reduced proliferation of intestinal epithelial cells and increased bacterial uptake in the intestinal epithelial cells [97]. Further, over-expression of *CAV2* or inhibition of miRNA-29a leads to activation of CDC24 (an important molecule that promotes the uptake of Salmonella into cells), suggesting a possible mechanistic pathway for Salmonella infection [97].

To date, there are no findings that describe a relationship between miRNA and *PTRF*. However, a recent study suggests that expression of *PTRF* may modulate the content of miRNA in extracellular vesicles secreted from prostate cancer cells [98].

### Summary of miRNA and caveolae related genes

The discovery and identification of miRNAs is beginning to provide understanding of the upstream regulatory mechanisms that regulate the expression of caveolae related genes. Some evidence is available for a relationship between miRNA and *CAV1* and *CAV2* (Table 2). However, the lack of the knowledge between miRNA and other caveolae related genes warrants further investigation.

**Table 2:** Relationship between miRNA and *CAV1* and *CAV2* in health and disease

miRNA	Target caveolae related gene	Changes observed and involvement in health and diseases
miRNA-133a	*CAV1*	miRNA-133a is up-regulated in head and neck squamous cell carcinoma and down-regulates *CAV1* [86]
miRNA-802	*CAV1*	miRNA-802 is increased and up-regulates potassium channel expression in kidney by down-regulating *CAV1* [87]
miRNA-103	*CAV1*	miRNA-103 is up-regulated in obese animals and associated with impaired glucose homeostasis by down-regulating *CAV1* [88]
miRNA-107	*CAV1*	miRNA-107 is up-regulated in obese animals and associated with impaired glucose homeostasis down-regulating *CAV1* [88]
miRNA-199a-5p	*CAV1*	miRNA-199a-5p is over-expressed in tissue fibrosis and pre-adipocytes, affects tissue fibrosis and proliferation of pre-adipocytes [91, 90]
miRNA-124	*CAV1*	miRNA-124 down-regulates *CAV1* and caveolae to prevent uptake of pathogens in kidney cells [93]
miRNA-192	*CAV1*	Exposure to DHA up-regulates miRNA-192 and down-regulates *CAV1* [94]
miRNA-199a-3p	*CAV2*	miRNA-199a-3p is up-regulated in breast cancer and down-regulates *CAV2* [95]
miRNA-218	*CAV2*	miRNA-128 is down-regulated in renal cell carcinoma and up-regulates *CAV2* [96]
miRNA-29a	*CAV2*	miRNA-29a is up-regulated following Salmonella infection and down-regulates *CAV2* [97]

### Perspective

Evidence suggests that environment and lifestyle factors may alter the epigenetic and miRNA profile in humans and contribute to disease [99]. As discussed above, caveolae related genes have been shown to play a role in the pathophysiology of various disease states, especially cancer. Although there is no evidence yet available that environmental changes or diets affect caveolae related genes epigenetically, growing evidence suggests that diet could affect the expression of miRNAs which will then affect the expression of caveolae related genes. Furthermore, it would be interesting to investigate the downstream effects of epigenetic changes to cellular physiology and pathophysiology. Currently, limited evidence is available on this aspect as most of the studies focus on the interaction of epigenetic changes to a particular caveolae related gene but not the downstream effects (eg: changes in cellular signaling mechanisms).

The involvement of mutations of the caveolae related genes may also contribute to changes in cellular physiology and pathophysiology. Mutations of *CAV1* and *PTRF* have been shown to be involved in congenital lipodystrophy [100, 101]. As yet there is no evidence available that epigenetic changes in expression of these genes causes lipodystrophic effects, this may suggest that epigenetic changes and mutations of caveolae related genes may predispose to different disease conditions through these two different pathways. Currently there are limited data concerning genetic mutations of *CAV2* or other caveolae associated genes.

Potentially, changes in the epigenetic status of caveolae related genes could be developed as a biomarker for diseases, in particular cancers. This could have several advantages. Firstly, DNA is more stable than RNA and secondly there are difficulties in differentiating between RNA from normal and tumor cells, meaning that there are advantages in using DNA as a biomarker over RNA [102, 103]. Furthermore, DNA promoter hypermethylation occurs uniquely in the CpG rich area in the promoter whereas genetic mutations can occur randomly within the coding and non-coding region, and promoter hypermethylation is an all-or-none event that can be detected easily with a single pair of primers. Lastly, minimally invasive methods can be used to collect samples (for example urine and plasma) that can then be used to detect epigenetic changes [104, 103]. Potentially, caveolae related genes that are silenced through epigenetic mechanisms may be a useful biomarker for diagnostic purposes in the future.

miRNA has been reported to be dysregulated in a variety of disease conditions. Even though the use of siRNA as a therapeutic target is being clinically trialed, the use of siRNA as a therapeutic target still poses some challenges. The high specificity, low toxicity, unique biogenesis and mechanism of action and the multiple targeting ability of miRNA provide advantages over siRNA. With the recognition of miRNA-caveolae related gene pathways in various disease states, miRNA may potentially be a useful tool for gene intervention. Nevertheless, a single miRNA is predicted to be able to bind to several hundreds of different mRNA [105]. Therefore, should a potential miRNA be developed as a therapeutic target, it would need to be targeted to a specific tissue to avoid unwanted effects which may occur in other tissues in the body. Thus, with the emerging knowledge of the role of miRNA in regulating caveolae related genes, modulating the expression of these key miRNAs could be a useful therapeutic tool, as caveolae related genes have been described to play important roles in health and disease.

## Conclusion

Caveolae related genes have been shown to play important roles in health and disease. Apart from genetic mutations, growing evidence suggests that epigenetic mechanisms may provide an upstream regulatory switch to control the expression of caveolae related genes hence contributing to disease conditions. Potentially, these two events may occur concurrently or exclusively to promote disease progression. Identification of epigenetic modifications may open new doors in biomarker and therapeutic target development to complement the current options that have been developed for genetic mutations. Much of the current evidence is focused on the changes in *CAV1* expression by methylation, acetylation and miRNA and less is known for *CAV2* and *PTRF*. Therefore, further studies are required to investigate whether altering the epigenetic state of these caveolae related genes can affect disease progression and if they can be used as biomarkers for disease identification.

## Abbreviations

CAV1: Caveolin-1
CAV2: Caveolin-2
CAV3: Caveolin-3
PTRF: Polymerase-1 and transcript release factor
5-AZA: 5-AZA-2′-deoxycytidine
TSA: Trichostatin-A
DNA: Deoxyribonucleic acid
RNA: Ribonucleic acid
miRNA: Micro-ribonucleic acid
CpGi: CpG island
HCC: Hepatocellular carcinoma
HNCC: Head and neck squamous cell carcinoma
RCC: Renal cell carcinoma
DHA: Docosahexaenoic acid
TGF-β: Transforming growth factor beta
ROMK: Renal outer medullary potassium
ER: Estrogen receptor
HBV: Hepatitis B Virus
IBC: Inflammatory breast cancer
kb: kilo base
mRNA: Messenger ribonucleic acid
